# Complete Genome Sequence of a Dimethyl Sulfide-Utilizing Bacterium, *Acinetobacter guillouiae* Strain 20B (NBRC 110550)

**DOI:** 10.1128/genomeA.01048-14

**Published:** 2014-10-16

**Authors:** LiiMien Yee, Akira Hosoyama, Shoko Ohji, Keiko Tsuchikane, Jun Shimodaira, Atsushi Yamazoe, Nobuyuki Fujita, Chiho Suzuki-Minakuchi, Hideaki Nojiri

**Affiliations:** a Biotechnology Research Center, University of Tokyo, Tokyo, Japan; b Biological Resource Center, National Institute of Technology and Evaluation, Tokyo, Japan

## Abstract

*Acinetobacter guillouiae* strain 20B can utilize dimethyl sulfide (DMS) as the sole sulfur source and degrade chloroethylenes. We report here the complete 4,648,418-bp genome sequence for this strain, which contains 4,367 predicted coding sequences (CDSs), including a well-characterized DMS degradative operon.

## GENOME ANNOUNCEMENT

The off-flavor compound dimethyl sulfide (DMS) is released into the atmosphere, especially from the ocean (1). *Acinetobacter guillouiae* (formerly designated as *Acinetobacter* sp.) strain 20B was isolated as a DMS degrader, which can utilize other volatile organic sulfur compounds, including dimethyl sulfoxide (DMSO) and dimethyl sulfone but is unable to utilize methanethiol as the sole sulfur source (2). It has been shown that strain 20B oxidizes these substrates via a DMS-oxidizing enzyme encoded by six open reading frames (ORFs) designated *dsoABCDEF*, which showed marked homology with the well-studied multicomponent phenol hydroxylases (2, 3). On the other hand, trichloroethylene (TCE) is a chlorinated solvent for dry cleaning and metal degreasing. Because of improper handling and disposal, TCE and its metabolites released into the environment may cause contamination of the biosphere and health problems (4). Previously, the DMS-oxidizing enzyme of strain 20B was found to degrade TCE (5).

The genomic DNA of strain 20B was sequenced by a combined method of shotgun sequencing using 454 GS FLX Titanium (Roche) and paired-end sequencing using HiSeq 1000 (Illumina). Two types of libraries were constructed, which generated sequences comprising 100,756,672 bp (248,914 reads, 21.7-fold coverage) and 577,068,962 bp (5,957,545 reads, 124.1-fold coverage), respectively. The sequences were assembled using Newbler version 2.6 (6). Gap closure was performed by Sanger sequencing of a fosmid library with inserts of approximately 32 kbp in pCC1FOS (Epicentre Biotechnologies, Madison, WI, USA).

The genome consists of a circular chromosome 4,648,418 bp in length with a GC content of 38.3%. The complete sequence of strain 20B genome was uploaded to the RAST server (http://rast.nmpdr.org) (7) to predict the ORFs, tRNAs, and rRNAs. The functional annotations of the predicted protein-coding genes were assigned based on UniProt, InterPro, HAMAP, and an in-house database composed of manually curated microbial genome sequences, as reported previously (8).

The analysis of average nucleotide identities based on BLAST (ANIb) (9) revealed that strain 20B is phylogenetically related to *A. guillouiae* (96.29%, accession no. BBLK00000000), *A. berezinae* (81.92%, accession no. BBLJ00000000), and *A. gerneri* (75.79%, accession no. BBLI00000000). This result, together with 16S rRNA gene homology, further confirms that strain 20B belongs to the species *A. guillouiae*.

Genes (AS4_16410-60) encoding a multicomponent monooxygenase, which is capable of oxidizing DMS and DMSO, were identified. The sequence showed a point mutation in the downstream region of *dso* genes, whose nucleotide sequence was registered in the DDBJ/EMBL/GenBank database (accession no. D85083). This may be due to spontaneous mutations. The DMSO utilization and TCE degradation abilities were reconfirmed by culturing with DSMO as the sole sulfur source and by biotransformation analysis using resting cells of strain 20B, respectively. Thus, strain 20B has potential use in the bioremediation of DMS-related organosulfur compounds and chloroethylenes. This genomic information may facilitate further studies using this strain for bioremediation of contaminated sites.

### Nucleotide sequence accession number.

The genome sequence of strain 20B genome sequence was deposited in the DDBJ/EMBL/GenBank databases under the accession number AP014630.
